# Prognostic value of postural hypotension in hospitalized patients with heart failure

**DOI:** 10.1038/s41598-022-06760-0

**Published:** 2022-02-18

**Authors:** Tsutomu Sunayama, Daichi Maeda, Yuya Matsue, Nobuyuki Kagiyama, Kentaro Jujo, Kazuya Saito, Kentaro Kamiya, Hiroshi Saito, Yuki Ogasawara, Emi Maekawa, Masaaki Konishi, Takeshi Kitai, Kentaro Iwata, Hiroshi Wada, Masaru Hiki, Taishi Dotare, Takatoshi Kasai, Hirofumi Nagamatsu, Tetsuya Ozawa, Katsuya Izawa, Shuhei Yamamoto, Naoki Aizawa, Ryusuke Yonezawa, Kazuhiro Oka, Shin-ichi Momomura, Tohru Minamino

**Affiliations:** 1 Department of Cardiovascular Biology and Medicine, Juntendo University Graduate School of Medicine, 2-1-1 Hongo, Bunkyo-ku, Tokyo, 113-8421 Japan; 2 Department of Cardiology, Osaka Medical and Pharmaceutical University, Takatsuki, Japan; 3 Cardiovascular Respiratory Sleep Medicine, Juntendo University Graduate School of Medicine, Tokyo, Japan; 4 Department of Cardiology, The Sakakibara Heart Institute of Okayama, Okayama, Japan; 5 Department of Digital Health and Telemedicine R&D, Juntendo University, Tokyo, Japan; 6 Department of Cardiovascular Biology and Medicine, Faculty of Medicine, Juntendo University, Tokyo, Japan; 7 Department of Cardiology, Nishiarai Heart Center Hospital, Tokyo, Japan; 8 Department of Rehabilitation, The Sakakibara Heart Institute of Okayama, Okayama, Japan; 9 Department of Rehabilitation, School of Allied Health Sciences, Kitasato University, Sagamihara, Japan; 10 Department of Rehabilitation, Kameda Medical Center, Kamogawa, Japan; 11 Department of Nursing, The Sakakibara Heart Institute of Okayama, Okayama, Japan; 12 Department of Cardiovascular Medicine, Kitasato University School of Medicine, Sagamihara, Japan; 13 Division of Cardiology, Yokohama City University Medical Center, Yokohama, Japan; 14 Department of Cardiovascular Medicine, National Cerebral and Cardiovascular Center, Osaka, Japan; 15 Department of Rehabilitation, Kobe City Medical Center General Hospital, Kobe, Japan; 16 Department of Cardiovascular Medicine, Saitama Medical Center, Jichi Medical University, Saitama, Japan; 17 Department of Cardiology, Tokai University School of Medicine, Isehara, Japan; 18 Department of Rehabilitation, Odawara Municipal Hospital, Odawara, Japan; 19 Department of Rehabilitation, Kasukabe Chuo General Hospital, Kasukabe, Japan; 20 Department of Rehabilitation, Shinshu University Hospital, Matsumoto, Japan; 21 Department of Cardiovascular Medicine, Nephrology and Neurology, University of the Ryukyus, Okinawa, Japan; 22 Rehabilitation Center, Kitasato University Medical Center, Kitamoto, Japan; 23 Department of Rehabilitation, Saitama Citizens Medical Center, Saitama, Japan; 24 Saitama Citizens Medical Center, Saitama, Japan; 25 Japan Agency for Medical Research and Development-Core Research for Evolutionary Medical Science and Technology (AMED-CREST), Japan Agency for Medical Research and Development, Tokyo, Japan

**Keywords:** Heart failure, Hypertension

## Abstract

Although postural hypotension (PH) is reportedly associated with mortality in the general population, the prognostic value for heart failure is unclear. This was a post-hoc analysis of FRAGILE-HF, a prospective multicenter observational study focusing on frailty in elderly patients with heart failure. Overall, 730 patients aged ≥ 65 years who were hospitalized with heart failure were enrolled. PH was defined by evaluating seated PH, and was defined as a fall of ≥ 20 mmHg in systolic and/or ≥ 10 mmHg in diastolic blood pressure within 3 min after transition from a supine to sitting position. The study endpoints were all-cause death and heart failure readmission at 1 year. Predictive variables for the presence of PH were also evaluated. PH was observed in 160 patients (21.9%). Patients with PH were more likely than those without PH to be male with a New York Heart Association classification of III/IV. Logistic regression analysis showed that male sex, severe heart failure symptoms, and lack of administration of angiotensin-converting enzyme inhibitors were independently associated with PH. PH was not associated with 1-year mortality, but was associated with a lower incidence of readmission after discharge after adjustment for other covariates. In conclusion, PH was associated with reduced risk of heart failure readmission but not with 1-year mortality in older patients with heart failure.

## Introduction

Heart failure is one of the most common diseases in the world and the number of patients is increasing mainly due to the aging society^1,2^. Despite improvements in the treatment of cardiovascular disease, the associated morbidity and mortality are unacceptably high. Along with the aging population, heart failure is a major cardiovascular problem associated with poor prognosis and high healthcare expenditure^3^.

Postural hypotension (PH) is a multifactorial syndrome that occurs when the blood pressure is notably decreased after moving to the upright posture from the supine or sitting position^4,5^. PH is prevalent in the elderly with a prevalence of approximately 20% in those aged ≥ 65 years^6^. Moreover, it has been shown that PH is associated with the incidence of heart failure and all-cause mortality in the general population^7–10^.

Heart failure and PH share older age, multiple comorbidities, and polypharmacy as common risk factors^11–13^. Previous studies have demonstrated that heart failure is an etiological factor for PH^14,15^, and that PH is a cause of heart failure^9,10^. However, very few studies with limited sample sizes have evaluated the clinical implications of PH in patients with heart failure, and of note, no study has addressed the prognostic value. The lack of information regarding the association between PH and prognosis in patients with heart failure is clinically relevant given that PH has been suggested as one of the reasons for not introducing or up-titrating, or even withdrawing guideline-directed medical therapy (GDMT)^16–19^. Indeed, a global survey regarding physician adherence to GDMT in patients with heart failure showed that hypotension is the second most common reason for intolerance to angiotensin-converting enzyme inhibitors (ACE-Is), the major reason for intolerance to angiotensin II receptor antagonists (ARBs), and the second most common reason for intolerance to beta blockers^20^. Therefore, this study aimed to determine the prevalence and prognostic value of PH in elderly patients with heart failure.

## Results

Of 730 patients (mean age 81.4 ± 7.7 years; 57.4% male), 160 (21.9%) were diagnosed with PH. Table 1 shows the baseline characteristics of patients with and without PH. Patients with PH were associated with male sex and a higher prevalence of New York Heart Association (NYHA) classification of III/IV. Notably, no difference was observed in age, baseline systolic/diastolic pressure, and the prescription of oral medications for heart failure. During the study period, 948 patients were registered to the FRAGILE-HF study from eight institutions that participated in the study focusing on PH, and 730 out of 948 (77.0%) patients who were registered from these institutions underwent PH evaluation. During the evaluation of PH, 13 (1.7%) patients experienced symptoms including light headache or dizziness. No patient reported severe symptoms, such as syncope, which mandate the evaluator to halt the test. No group difference was found in the prevalence of symptoms related to seated PH evaluation (2.5% and 1.6% for those with and without PH, respectively; *P* = 0.500).

**Table 1:** Baseline characteristics of the study cohort.

Variables	Non-PH group	PH group	*P* value
	n = 570	n = 160	
Age (years)	82 (76–87)	82 (75–86)	0.203
Male sex, n (%)	298 (52.3)	101 (63.1)	0.019
Body mass index (kg/m^2^)	22 ± 4	21 ± 4	0.291
NYHA Class III/IV, n (%)	56 (9.8)	26 (16.2)	0.033
Systolic blood pressure (mmHg)	114 ± 16	116 ± 16	0.204
Diastolic blood pressure (mmHg)	62 ± 10	63 ± 11	0.328
Heart rate (bpm)	70 ± 14	71 ± 14	0.162
LVEF (%)	47.4 ± 16.5	48.2 ± 15.5	0.625
**Heart failure phenotypes, n (%)**			0.355
HFrEF	216 (38.2)	52 (33.1)	
HFmrEF	92 (16.3)	32 (20.4)	
HFpEF	258 (45.6)	73 (46.5)	
**History of heart failure, n (%)**			0.943
None	255 (44.7)	71 (44.4)	
< 1.5 years	76 (13.3)	23 (14.4)	
> 1.5 years	239 (41.9)	66 (41.2)	
**Comorbidities, n (%)**			
Atrial fibrillation	247 (43.3)	80 (50.0)	0.159
Coronary artery disease	221 (38.8)	61 (38.1)	0.955
COPD	64 (11.2)	22 (13.8)	0.462
Diabetes	199 (34.9)	54 (33.8)	0.858
Hypertension	411 (72.1)	122 (76.2)	0.346
**Prescription at discharge, n (%)**			
ACE-Is/ARBs	359 (63.0)	87 (54.4)	0.060
Beta blockers	416 (73.0)	117 (73.1)	0.999
MRAs	38 (6.7)	13 (8.1)	0.643
Loop diuretics	313 (54.9)	88 (55.0)	0.999
**Laboratory data at discharge**			
White blood cells (/μL)	5400 (4360–6700)	5450 (4590–7030)	0.217
Hemoglobin (g/dL)	11.8 ± 2.1	11.7 ± 2.1	0.640
Sodium (mEq/L)	139 ± 4	140 ± 4	0.438
Albumin (g/dL)	3.4 ± 0.5	3.4 ± 0.5	0.153
Creatinine (mg/dL)	1.4 ± 0.7	1.4 ± 0.6	0.520
eGFR (mL/min/1.73m^2^)	52 ± 22	51 ± 20	0.589
BUN (mg/dL)	28 (21–37)	27 (20–36)	0.234
C-reactive protein (mg/dL)	0.28 (0.11–0.83)	0.23 (0.10–0.90)	0.768
BNP (pg/mL)	244 [116–464]	212 [120–453]	0.745

Continuous variables are expressed as the mean ± standard deviation or median [25–75%].

*ACE-I* angiotensin-converting enzyme inhibitors, *ARB* angiotensin II receptor antagonists, *BNP* brain natriuretic peptide, *BUN* blood urea nitrogen, *COPD* chronic obstructive pulmonary disease, *eGFR* estimated glomerular filtration rate, *LVEF* left ventricular ejection fraction, *MRA* mineralocorticoid receptor antagonists, *NYHA* New York Heart Association, *PH* postural hypotension.

The results of the univariate and multivariable logistic analyses of the presence of PH are described in Table 2. After including all variables with *P* < 0.10 in the univariate model, male sex, NYHA classification of III/IV, and the prescription of ACE-Is remained significant predictors of PH. In the sensitivity analysis, we included age and a history of diabetes in the multivariable analysis since these factors were reported to be strongly associated with PH^21,22^. The result was consistent even after adding these two factors (Table 2).

**Table 2:** Logistic analysis for the presence of postural hypotension.

	Unadjusted model			Adjusted model 1			Adjusted model 2		
	Odds ratio	95% CI	*P* value	Odds ratio	95% CI	*P* value	Odds ratio	95% CI	*P* value
Age (years)	0.99	0.97–1.01	0.263				0.98	0.96–1.01	0.146
Male sex	1.56	1.09–2.24	0.015	1.69	1.17–2.45	0.006	1.61	1.10–2.36	0.014
Body mass index (kg/m^2^)	0.98	0.93–1.02	0.291						
NYHA Class III/IV	1.78	1.08–2.94	0.024	1.84	1.09–3.09	0.022	1.89	1.12–3.19	0.017
Systolic blood pressure (mmHg)	1.01	1.00–1.02	0.204						
Diastolic blood pressure (mmHg)	1.01	0.99–1.03	0.328						
Heart rate (bpm)	1.01	1.00–1.02	0.163						
LVEF (%)	1.00	0.99–1.01	0.625						
Atrial fibrillation	1.31	0.92–1.86	0.135						
Diabetes	0.95	0.66–1.38	0.785				0.94	0.64–1.38	0.756
Hypertension	1.24	0.83–1.87	0.297						
ACE-Is	0.62	0.41–0.93	0.022	0.62	0.41–0.93	0.023	0.59	0.38–0.90	0.013
ARBs	1.10	0.75–1.60	0.633						
Beta blockers	1.01	0.68–1.50	0.971						
MRAs	1.24	0.64–2.39	0.523						
Loop diuretics	1.00	0.71–1.43	0.984						
*White blood cells (/μL)	1.42	0.82–2.47	0.210						
Hemoglobin (g/dL)	0.98	0.90–1.07	0.640						
Sodium (mEq/L)	1.02	0.97–1.07	0.437						
Creatinine (mg/dL)	1.09	0.84–1.42	0.520						
C-reactive protein (mg/dL)	1.09	0.99–1.21	0.088	1.08	0.98–1.20	0.140	1.09	0.98–1.21	0.097
*BNP (pg/mL)	1.00	0.84–1.19	0.999						

*ACE-I* angiotensin-converting enzyme inhibitor, *ARB* angiotensin II receptor antagonist, *BNP* brain natriuretic peptide, *CI* confidence interval, *LVEF* left ventricular ejection fraction, *MRA* mineralocorticoid receptor antagonist, *NYHA* New York Heart Association, *PH* postural hypotension.

*Variables were transformed into the logarithmic scale.

**Figure 1:**
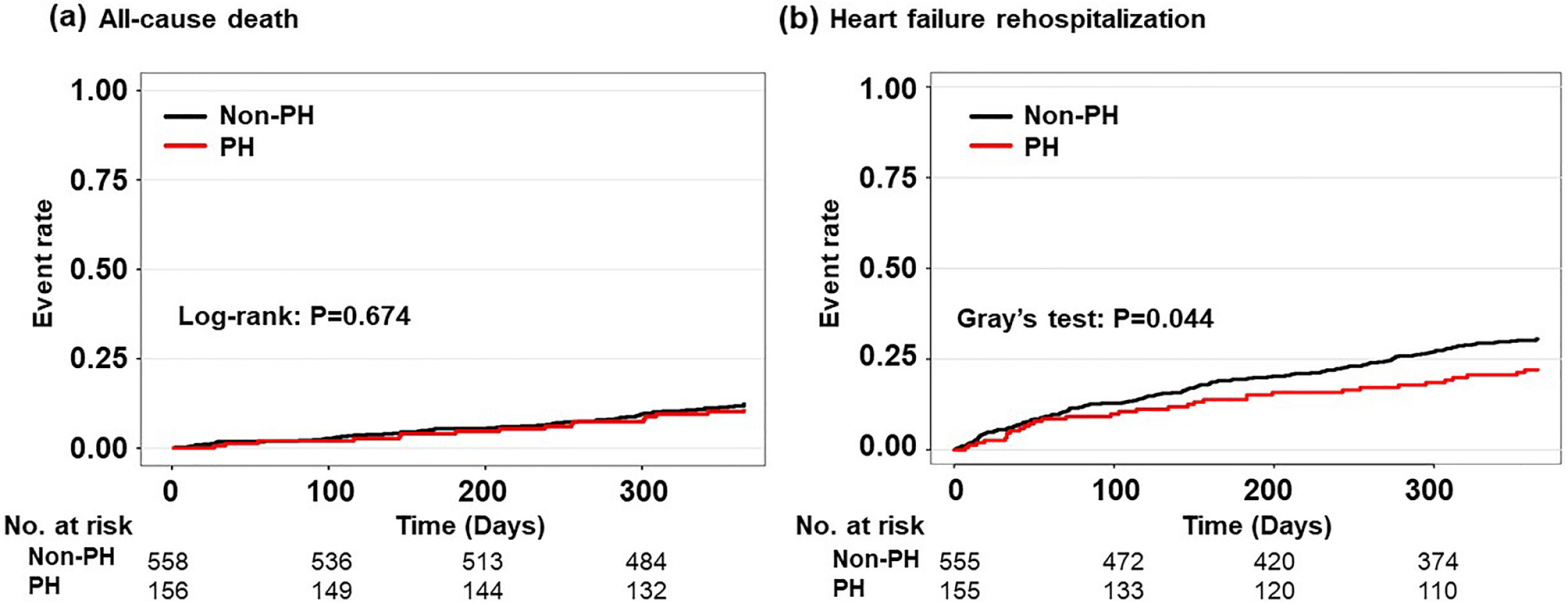
Kaplan–Meier analysis of all-cause death and heart failure readmission. Postural hypotension (PH) was not associated with all-cause mortality (**a**), but was significantly associated with a lower incidence of heart failure readmission (**b**).

Of the study patients, 714 (97.8%) were followed-up for 1-year mortality and 710 (97.3%) for heart failure readmission. During the 1-year follow-up, 87 deaths (11.9%) and 200 cases of heart failure readmission (27.4%) were observed. Kaplan–Meier survival analysis followed by the log-rank test showed no group difference in 1-year mortality (Fig. 1a). Contrastingly, patients with PH had a lower incidence of heart failure readmission than those without PH (Fig. 1b). Cox proportional hazard regression analysis revealed that the presence of PH was not associated with 1-year mortality after adjusting for the Meta-analysis Global Group in Chronic Heart Failure (MAGGIC) risk score and log-transformed brain natriuretic peptide (BNP) (hazard ratio, 0.94; 95% confidence interval 0.55–1.63; *P* = 0.835) (Table 3). Adjusted Fine and Gray competing risk analyses indicated that PH was significantly associated with a lower rate of heart failure readmission after adjusting for potential confounding factors (hazard ratio, 0.63; 95% confidence interval 0.42–0.96; *P* = 0.030) (Table 3).

**Table 3:** Cox proportional hazard analysis of 1-year mortality and heart failure readmission.

Groups	All-cause death						Heart failure readmission					
	Unadjusted Cox model			*Adjusted Cox model			Unadjusted Fine-Gray model			**Adjusted Fine-Gray model		
	HR	95% CI	*P* value	HR	95% CI	*P* value	HR	95% CI	*P* value	HR	95% CI	*P* value
Non-PH group	1 (Reference)			1 (Reference)			1 (Reference)			1 (Reference)		
PH group	0.89	0.53–1.52	0.677	0.94	0.55–1.63	0.835	0.69	0.47–0.99	0.048	0.63	0.42–0.96	0.030

*CI* confidence interval, *HR* hazard ratio, *PH* postural hypotension.

*Adjusted for the Meta-analysis Global Group in Chronic Heart Failure risk score and log–transformed brain natriuretic peptide.

**Adjusted for age, sex, New York Heart Association classification of III/IV, systolic blood pressure, hemoglobin, albumin, estimated glomerular filtration rate, sodium, log-transformed brain natriuretic peptide, left ventricular ejection fraction, history of heart failure, atrial fibrillation, coronary artery disease, diabetes, angiotensin-converting enzyme inhibitor/angiotensin II receptor antagonist, beta blocker, and mineralocorticoid receptor antagonist.

## Discussion

To the best of our knowledge, this is the first study to prospectively investigate the prognostic value of PH in patients with heart failure. We demonstrated that (1) 22% of the study population was affected by PH; (2) PH was associated with male sex, high NYHA classification, and not taking ACE-Is; and (3) patients with PH had a lower risk of heart failure readmission after discharge and PH was not associated with all-cause death.

In our study, 22% of the patients with heart failure had PH. Previous reports indicated that the prevalence of PH in patients with heart failure varied widely, ranging from 8 to 83%^12,23–25^. The reason for this variability may be attributed to differences in the baseline patient characteristics (i.e., age, sex, medication, or severity of heart failure) and diagnostic criteria for PH. For instance, a previous study evaluating 108 patients with acute decompensated heart failure reported that 50% of the study population was affected by PH, which was a higher prevalence than that in our study^12^. In the same study, 49% of the participants were categorized as NYHA classification III/IV^12^, whereas only 11% of our participants were categorized as such. Moreover, previous studies regarding PH in patients with heart failure evaluated very limited numbers of patients (24–180 patients), which may have led to variability in the reported prevalence^15^. The current study is one of the largest studies to evaluate the prevalence of PH in patients with heart failure.

We found that the use of ACE-Is was related to a lower prevalence of PH, which may be opposite to the expectation. However, a previous study that evaluated middle-aged patients with hypertension demonstrated that use of ACE-Is was associated with the absence of PH^26^, which is in line with our results. The mechanism behind this association is unclear; however, the nephroprotective effect of ACE-Is has been suggested as a possible factor^26^. We also found that a higher NYHA classification and male sex were associated with PH. This finding is supported by a previous study of patients with acute decompensated heart failure, which found that an NYHA classification of III/IV was significantly associated with seated PH^12^. This association could be partially explained by the fact that a higher NYHA classification relates to longer bed rest and a higher prevalence of multiple chronic comorbidities, both of which are risk factors for PH^11,12^. Regarding the association between male sex and PH, there are no available data on the association between sex and PH in patients with heart failure. That may be because of differences in baseline characteristics between males and females, although the underlying mechanisms are unclear and should be investigated in future studies.

Although the mechanism underlying the association between a low heart failure hospitalization rate and PH is unclear, one hypothesis is that PH could be an indicator of the well-decongestive status in patients with heart failure. Dehydration is a well-known cause of PH^27^, and a position statement proposed by the European Society of Cardiology considered PH a sign of the euvolemic status in patients with heart failure^28^. Since we did not obtain data regarding changes in body weight, BNP, diuretic response, hemoconcentration, or echocardiographic parameters at admission, we could not evaluate our hypothesis that PH before discharge is associated with decongestion status. Further studies are needed to clarify the pathophysiological mechanism behind PH in patients with heart failure.

Regarding the association between PH and mortality, previous studies focusing on general population cohorts revealed that PH was significantly associated with high mortality^7,29,30^. Possible reasons for the relationship between PH and poor prognosis include autonomic dysfunction^4,31^ and reduced coronary flow^32^. However, a post-hoc analysis of the SPRINT trial involving patients at high risk of cardiovascular disease showed no significant association between PH and mortality^33^. The results were consistent with our current finding that the presence of PH was not associated with 1-year mortality in patients with heart failure. The discrepancy regarding the impact of PH on mortality across studies may be attributable to the difference in study populations or prescription rate of drugs associated with PH, such as renin-angiotensin system inhibitors.

Our finding of the lack of association between PH and unfavorable prognosis including mortality is relevant given that the drugs recognized as GDMT for heart failure exert blood pressure-lowering effects and may cause hypotension. This may prompt clinicians to withdraw or down-titrate GDMT. However, previous studies have clearly demonstrated that down-titration of GDMT is associated with poor prognosis^34–36^. Moreover, such drugs may not affect the risk of PH^37^. Therefore, we believe that dose reduction or withdrawal of GDMT in patients with heart failure based on the consideration of PH alone is not justified, and prospective large-scale randomized clinical studies should aim to address this issue in the future.

This study has several limitations. First, we did not obtain data on the inter- and intra-rater reliability of PH measurement. PH was evaluated at eight out of 15 hospitals by different measurers; thus, inter-observer bias cannot be excluded. Second, the seated PH, rather than standardized tests for PH including the tilt test, was used to define PH in our study. Since the clinical validity of seated PH might be lower than that of other well-validated tests for PH, it may have impacted our study results and conclusions. However, Shaw et al. compared the passive seated orthostatic stress test to the passive head-up tilt testing and found no significant difference in blood pressure and other hemodynamic variables^38^. Moreover, seated PH may be more generalizable with regards to safety compared to active standing given that patients with heart failure are old and likely frail. However, we should emphasize that the gold standard of evaluation for PH suggested in the guidelines is head-up tilt testing, and this may have impacted our findings and conclusions^39^. It should also be noted that we only evaluated PH at 1 and 3 min after passive seating; some patients subsequently experienced significant decreases in blood pressure or symptoms^40^, and this may have impacted our results. For instance, changes in blood pressure were measured until 5 min^12,41^ or 10 min after postural change in other studies that focused on PH. Third, we did not obtain data regarding the causes of readmission other than heart failure, even though the majority of readmissions after hospitalization for heart failure are due to non-cardiovascular causes. Fourth, since we only included Japanese patients, it is unclear whether the findings of this study are applicable to Western populations. Lastly, patients who could not walk unaided were excluded from our study. Since these populations are at a high risk of developing PH, the generalizability of our results is limited.

In conclusion, our findings showed that PH was observed in 22% of elderly hospitalized patients with heart failure. The presence of PH was not associated with 1-year mortality, but was associated with a lower risk of heart failure readmission. Our findings imply that PH is not necessarily a marker of poor prognosis; therefore, the presence of PH does not justify the withdrawal or down-titration of drugs that have been confirmed to improve the prognosis of patients with heart failure.

## Methods

### Study design and participants

This was a sub-analysis of the FRAGILE-HF study, a prospective and multicenter study (six universities and nine non-university hospitals) with the primary objective of evaluating multi-domain frailty in elderly patients with heart failure. The full details of the study design and main results have been published previously^42^. All consecutive 1332 hospitalized patients with decompensated heart failure aged ≥ 65 years who could ambulate at discharge were registered. Decompensated heart failure was defined using the Framingham criteria^43^.

The exclusion criteria were as follows: (1) a history of heart transplantation or left ventricular assist device implantation; (2) undergoing chronic peritoneal dialysis or hemodialysis therapy; (3)a diagnosis of acute myocarditis; (4) lack of data on BNP or N-terminal-pro BNP values; and (5) a BNP level < 100 pg/mL or N-terminal-pro BNP level < 300 pg/mL at admission. Physical examination, blood sampling, and echocardiography were performed before discharge in the clinically compensated phase, and medication information was also obtained at discharge.

All participants were informed of the study and it was explained that they were free to opt-out of participation whenever they wanted. Written informed consent was not required under the Ethical Guidelines for Medical and Health Research Involving Human Subjects, issued by the Japanese Ministry of Health, Labor, and Welfare, because the current study was an observational study without invasive procedures. The study complied with the Declaration of Helsinki and the Japanese ethical guidelines for medical and health research involving human subjects. This study protocol was approved by the research ethics committee of each participating hospital (the Sakakibara Heart Institute of Okayama, Research ethics committee [no approval number, approval date: August 18th, 2016], Juntendo University Graduate School of Medicine, Research ethics committee [approval number: 16-150]; Nishiarai Heart Center Hospital, Research ethics committee [approval number: 2016-03]; Kitasato University, Research ethics committee [approval number: B16-107]; Kameda Medical Center, Research ethics committee [approval number: 16-080]; Yokohama City University Medical Center, Research ethics committee [approval number: B161000019]; Kobe City Medical Center General Hospital, Research ethics committee [approval number: zn170114]; Saitama Medical Center, Research ethics committee [approval number: S16-035]; Tokai University School of Medicine, Research ethics committee [approval number: 16R122]; Odawara Municipal Hospital, Research ethics committee [approval number: 2016-01]; Kasukabe Chuo General Hospital, Research ethics committee [approval number: 1702-4]; Shinshu University Hospital, Research ethics committee [approval number: 3565]; University of the Ryukyus, Research ethics committee [approval number: 365]; Kitasato University Medical Center, Research ethics committee [approval number: 28-37]; Saitama Citizens Medical Center, Research ethics committee [no approval number, approval date: December 26th, 2016]). Detailed study information was published on the publicly available University Hospital Information Network (UMIN-CTR, unique identifier: UMIN000023929) before the first patient was enrolled.

### Measurement of PH

**Figure 2:**
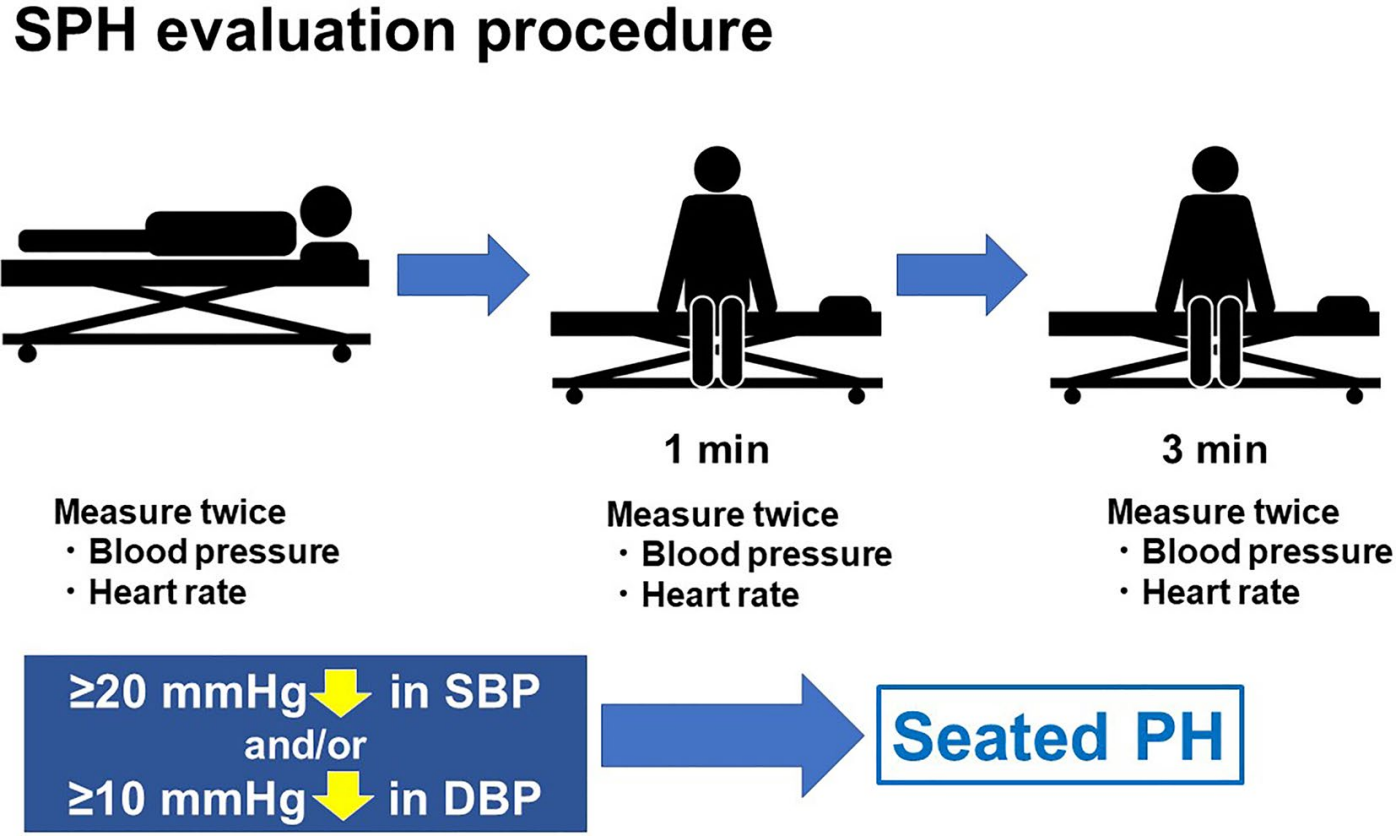
Assessment of seated postural hypotension. After 5 min of rest in the supine position, blood pressure and heart rate were measured at baseline. The measurements were repeated 1 and 3 min after passive seating in the bed with the legs bent at the knee and hanging over the side of the bed. All blood pressure and heart rate measurements were performed twice, and the mean value was used. Postural hypotension was defined as a decrease of ≥ 20 mmHg in systolic blood pressure and/or ≥ 10 mmHg in diastolic blood pressure. *DBP* diastolic blood pressure, *SBP* systolic blood pressure, *SPH* seated postural hypotension.

The evaluation of PH was optional and not required for all participating institutions of the FRAGILE-HF study. In total, eight of 15 hospitals decided to take part in this optional study before we started the FRAGILE-HF. PH was evaluated when intravenous therapies for heart failure were finished and patients were considered to be in a clinically compensated state of heart failure. As we focused on elderly patients, who may not be able to stand upright for an extended period, the passive seated orthostatic stress test^15^ was used for evaluating PH in the current study for safety reasons (Fig. 2). Patients were not allowed to consume caffeine or alcohol for 12 h or eat anything for 1 h before PH evaluation. After 5 min of rest in the supine position, blood pressure and heart rate were recorded as baseline. The measurements were repeated 1 and 3 min after passive seating in a bed with the legs bent at the knee and hanging over the side of the bed, which was bent at an angle of 90°. All blood pressure and heart rate measurements were performed twice, and the mean value was used. PH was defined as a decrease of ≥ 20 mmHg in systolic and/or ≥ 10 mmHg in diastolic blood pressure^44^. When patients met this criterion at least once (at 1 min and/or 3 min after the 5-min rest period), PH was diagnosed.

### Study endpoint

The primary endpoint of the current study was all-cause mortality at 1 year, and the secondary endpoint was readmission due to worsening heart failure. After discharge, most patients were followed-up in outpatient clinics at least every 3 months and also according to their medical needs. For those who did not undergo follow-up in clinics, prognostic data were obtained via telephone interviews and medical records were obtained from other medical facilities where the patient received care or from the family. Heart failure readmission was defined according to the criteria described in the American College of Cardiology/American Heart Association Key Data Elements and Definitions for Cardiovascular Endpoint Events in Clinical Trials^45^.

### Statistical analysis

Categorical variables are represented as numbers and percentages. Continuous variables are reported as the mean ± standard deviation if normally distributed, and as the median with interquartile range if non-normally distributed. Non-normally distributed variables were transformed into the logarithmic scale for further analyses. The Student’s t-test or Mann–Whitney U test for continuous variables and the chi-squared or Fisher’s exact tests for categorical variables were used to compare group differences.

Factors associated with PH were assessed using univariate and multivariable logistic regression analysis. Variables with *P* < 0.10 in the unadjusted model were selected for inclusion in the multivariable model. Cumulative incidence curves for all-cause death were calculated using Kaplan–Meier estimates. For heart failure readmission, cumulative incidence curves were generated using a Fine–Gray competing risk regression analysis, with death that was not the consequence of heart failure readmission included as a competing risk. A Cox proportional hazards model was used to investigate the association between PH and 1-year mortality. To assess potential prognostic factors for all-cause mortality, the MAGGIC risk score^46^ and log-transformed BNP were entered into the adjustment model^42,47–50^. The MAGGIC risk score is a well-established prognostic score for the assessment of mortality in various cohorts including Japanese populations, and adding BNP values at discharge to the MAGGIC risk score improves prognostic values^46^. Furthermore, the cumulative incidence of heart failure readmission stratified by the GDMT group was compared using the Gray test^51^. For the multivariable competing risk model, the following variables were included as the adjustment variables: age; sex; NYHA functional classification of III/IV; systolic blood pressure; hemoglobin levels; albumin levels; estimated glomerular filtration rate; natrium; log-transformed BNP value; left ventricular ejection fraction; history of heart failure; atrial fibrillation; coronary artery disease; diabetes; and prescription of ACE-Is/ARBs, beta blockers, and mineralocorticoid receptor antagonists.

Two-tailed *P* values < 0.05 were considered statistically significant. All analyses were performed using R version 3.5.2 (R Foundation for Statistical Computing, Vienna, Austria; ISBN 3–900 051–07-0, URL http://www.R-project.org).

## Data Availability

The datasets generated during and/or analyzed during the current study are available from the corresponding author upon reasonable request.
